# Effect of corticosteroids combined with cyclophosphamide or mycophenolate mofetil therapy for IgA nephropathy with stage 3 or 4 chronic kidney disease: A retrospective cohort study

**DOI:** 10.3389/fphar.2022.946165

**Published:** 2022-08-31

**Authors:** Qing Jia, Feng Ma, Jin Zhao, Xiaoxia Yang, Ruiling Sun, Rong Li, Shiren Sun

**Affiliations:** ^1^ Department of Nephrology, Xijing Hospital, Fourth Military Medical University, Xi’an, Shaanxi, China; ^2^ Xi’an Jiao Tong University-affiliated Honghui Hospital, Xi’an, Shaanxi, China

**Keywords:** IgA nephropathy, renal insufficiency, corticosteroids, mycophenolate mofetil, cyclophosphamide

## Abstract

**Background:** To determine the safety and efficacy of corticosteroids (CS) combined with cyclophosphamide (CTX), compared with CS combined with mycophenolate mofetil (MMF) for IgA nephropathy (IgAN) patients with stage 3 and 4 CKD and proteinuria ≥1.0 g/24 h in a 10-year real-world study.

**Methods:** We recruited 296 IgAN patients with renal insufficiency and proteinuria ≥1.0 g/24 h who received uncontrolled supportive care (USC) (*n* = 44), CS + CTX therapy (n = 164) and CS + MMF therapy (n = 88) in Xijing Hospital from July 2008 to December 2019. The combined event was defined as a ≥50% decrease in eGFR, ESRD, or death.

**Results:** The median of the follow-up period was 39.3 months. One hundred and twenty-five patients experienced the combined event, 65.9, 37.8, and 38.6% in the USC, CS + CTX, and CS + MMF group, respectively. In multivariate Cox regression analyses, CS combined with CTX (HR = 0.457, 95% CI 0.238-0.878, *p* = 0.019) significantly reduced the incidence of the combined event, whereas CS + MMF (HR = 0.523, 95% CI 0.246-1.109, *p* = 0.091) did not reduce the risk of the combined event, compared with USC. The incidence of pneumonia and death due to infection in the CS + MMF group was higher than other two groups.

**Conclusion:** Compared with USC and CS + MMF therapy, CS + CTX therapy was more safety and possibly more effective. The results need to be further confirmed by large randomized controlled studies.

## Introduction

IgA nephropathy (IgAN) is one of the most common primary glomerular diseases worldwide (Wyatt and Julian, 2013) and is recognized as an autoimmune disease (Rodrigues et al., 2017). Also, IgAN is an important cause of end-stage renal disease (ESRD) (Lai et al., 2016). About 20% of IgAN patients present with stage 3 or 4 chronic kidney disease (CKD) at renal biopsy in China (Lv et al., 2008; Le et al., 2012), nearly 80% of IgAN patients with renal insufficiency of diagnosis progress to ESRD within 7–10 years (Lv et al., 2008).

Unfortunately, due to the pathogenesis of IgAN is incompletely clear (Rodrigues et al., 2017), the best option of treatment for IgAN patients is uncertain, especially for those with renal insufficiency. Two well-designed randomized clinical trials (RCT) (Lv et al., 2017; Rauen et al., 2018) did not show the efficacy of immunosuppressive therapy for IgAN patients with renal insufficiency. Moreover, some studies reported that the incidence of immunosuppressive therapy related adverse events was increased in IgAN patients with renal insufficiency (Pozzi et al., 2016; D'Amico et al., 1993; Pozzi et al., 2010). However, many retrospective studies (Roccatello et al., 2000; Rasche et al., 2004; Moriyama et al., 2011; Tan et al., 2018; Beck et al., 2022) and other RCT studies (Ballardie and Roberts, 2002; Pozzi et al., 2013) reported that immunosuppressive therapy was effective in postponing the renal function progression in IgAN patients with renal insufficiency without increasement of adverse events. Our previous publication also showed that IgAN patients with renal insufficiency had a better renal prognosis after received immunosuppressive therapy, and the adverse effects were tolerable (Yang et al., 2021). Thus, immunosuppressive therapy may be a potentially effective treatment option for IgAN patients with renal insufficiency. However, this undetermined conclusion was drawn from studies that did not include IgAN patients with stage 4 CKD(19) or from small sample sizes studies (Ballardie and Roberts, 2002; Pozzi et al., 2013), due to the paucity of direct evidence focused on IgAN patients with stage 3 or 4 CKD. Therefore, the Kidney disease: Improving Global Outcomes (KDIGO) guideline (2021) suggests clinicians should adequately evaluate the treatment-emergent toxicity of the use of immunosuppressive therapy in IgAN patients with eGFR <50 ml/min per 1.73 m^2^ and persistent proteinuria ≥0.75–1.0 g/24 h after ≥3 months maximal supportive care (Rovin et al., 2021). Preferably, the guideline suggests these patients the opportunity to take part in a therapeutic clinical trial.

Evidence shows that clinicians tend to give steroid-based immunosuppressive therapy for IgAN patients with renal insufficiency due to poor prognosis after supportive care therapy (Coppo et al., 2014), despite no recommended guideline. Immunosuppressants included cyclophosphamide (CTX) and mycophenolate mofetil (MMF) are often used as glucocorticoid-sparing agents (Rovin et al., 2021) for IgAN patients. In our center, low-dose corticosteroid (CS) combined with CTX and CS combined with MMF were the most common therapies for IgAN patients with renal insufficiency. Our previous study showed that compared with supportive care and CS monotherapy, low-dose CS combined with CTX therapy could improve renal survival for IgAN patients with renal insufficiency with no difference in incidence of adverse events (Ma et al., 2020). Another previous study we conducted showed that low-dose CS combined with MMF therapy effectively improved renal prognosis than supportive care therapy, but increased the risk of severe pneumonia and related death (Zhao et al., 2021). Both two studies revealed that low-dose steroid-based immunosuppressive therapy may improve renal survival compared with supportive therapy and CS monotherapy in IgAN patients with renal insufficiency. Nevertheless, which immunosuppressive therapy is the better option for IgAN patients with renal insufficiency is unknown. Herein, we aim to evaluate the efficacy and safety of low-dose CS combined with CTX or MMF in IgAN patients with renal insufficiency in a 10-year real-world cohort study.

## Materials and methods

### Study population

**FIGURE 1:**
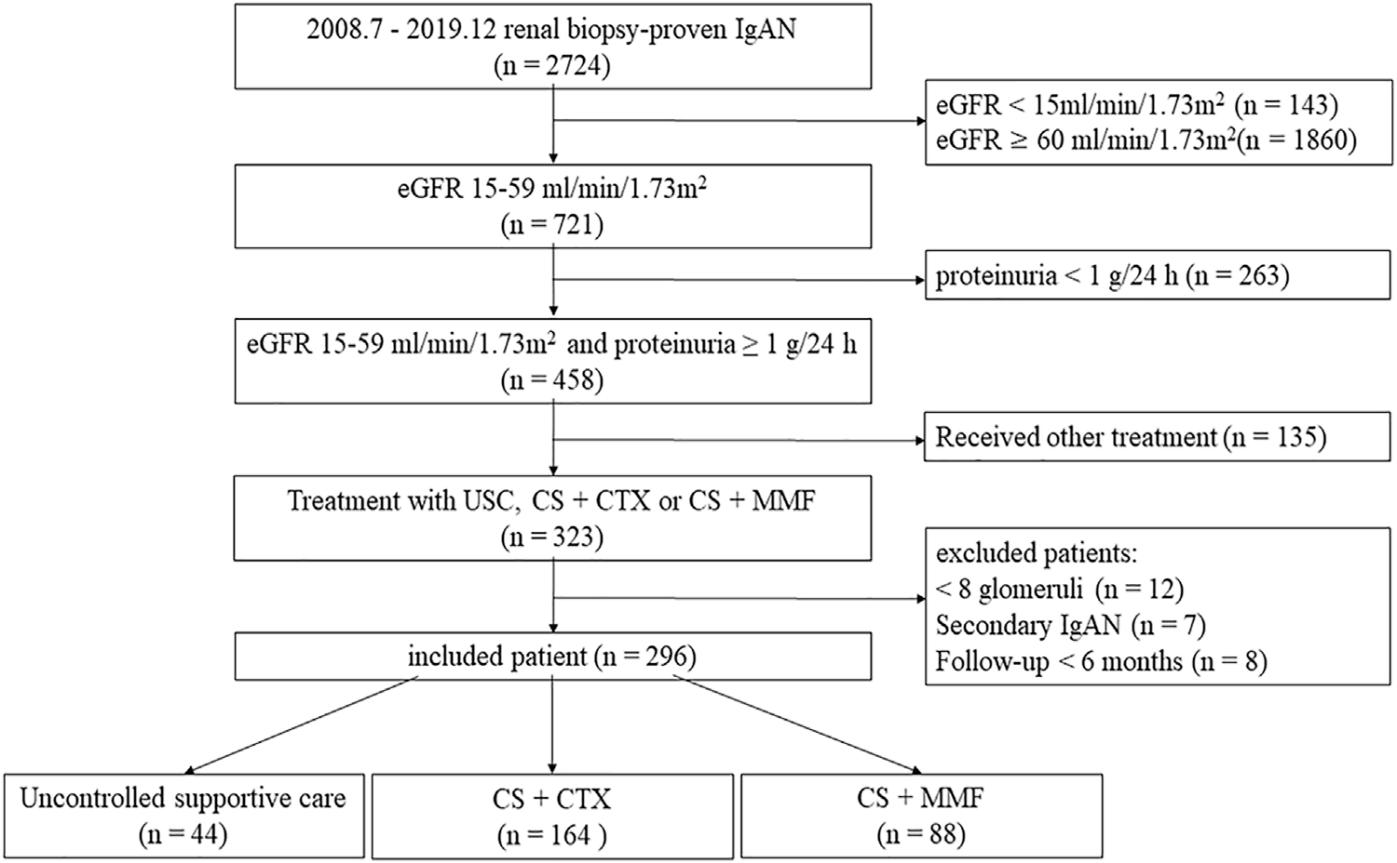
Inclusion flowchart. eGFR, estimated glomerular filtration rate; UCS, uncontrolled supportive care; CS, corticosteroids; CTX, cyclophosphamide; MMF, mycophenolate mofetil.

We recruited 2724 renal biopsy-proven IgAN patients in Xijing Hospital from July 2008 to December 2019. We extracted i) patients with eGFR 15–60 ml/min per 1.73 m^2^ and proteinuria ≥1.0 g/24 h, ii) patients who received uncontrolled supportive care therapy, CS combined with CTX, or CS combined with MMF. We excluded: i) patients with <8 glomeruli in biopsy specimens (*n* = 12), ii) secondary IgAN, such as purpura nephritis, lupus nephritis, and hepatitis B-associated glomerulonephritis (*n* = 7) and iii) patients with less than 6 months follow-up period unless they meet the end-points (*n* = 8). Two hundred and sixty-seven patients were finally included in our study (Figure 1). These patients were divided into three groups, an uncontrolled supportive care (USC) group (n = 44), a glucocorticoid plus cyclophosphamide (CS + CTX) group (n = 164) and a glucocorticoid plus mycophenolate mofetil (CS + MMF) group (n = 88). The study was approved by the ethics committee of Xijing Hospital (ethical number: KY20213027-1).

### Data collection

We collected the baseline demographic, clinical and pathological characteristics at the time of renal biopsy. Demographic characteristics included age and gender. Clinical characteristics included systolic blood pressure (SBP), diastolic blood pressure (DBP), gross hematuria, microscopic hematuria, 24 h urinary protein excretion, serum creatinine, estimated glomerular filtration rate (eGFR), serum albumin, serum IgA, complement C3 and the use of renin-angiotensin system blockers (RASB). Pathological characteristics were reviewed by an experienced pathologist and indicated by the updated Oxford Classification of IgAN (MEST-C). We also collected these patients’ follow-up data such as follow-up period, time-average proteinuria (TA-P), time-average MAP (TA-MAP), eGFR, renal outcomes, and adverse events. Adverse events include pneumonia, leukopenia (leukocyte count <4000/µL), anemia, stomach discomfort, hepatic injury (alanine aminotransferase (ALT) > 50 IU/ml), diabetes mellitus, tumor, and death. The frequency of follow-up was at least 6 months. All patients were followed up until March 2022.

### Treatments

All patients in three groups received RASB therapy if they can tolerate it. In CS + CTX and CS + MMF groups, patients receive prednisolone 30–40 mg/day for 8 weeks, followed by a monthly reduction of 5.0 mg, tapered to 10 mg for 6 months. Cyclophosphamide was given to patients intravenously 0.6–1.0 g/month for 6 months or orally 50 mg/day for 5 months. Mycophenolate mofetil was given to patients 1.0–1.5 g/day decided by the body weight for 6 months, and reduced to 0.5–0.75 g/day for 6 months, and then gradually decrease the dosage until it is stopped. The choice of therapeutic agents is based on the experience of the chief doctor. All patients who received CTX were instructed to have copious fluid intake, but not use mercaptoethane sulfonate (Mesna) regular.

### Definitions

ESRD was defined as eGFR <15 ml/min per 1.73 m^2^, starting chronic dialysis (hemodialysis or peritoneal dialysis) or receiving a kidney transplant. GFR was estimated by the Chronic Kidney disease Epidemiology Collaboration (CKD-EPI) formula. CKD was defined and classified according to 2021 KDIGO clinical practice guideline. Kidney lesions were classified by the updated Oxford Classification of IgAN. The TA-P was an average calculated from all proteinuria measurements taken during each patient follow-up. The TA-MP was an average calculated from all MAP measurements taken during each patient follow-up. The combined event referred to ESRD, a ≥50% decrease in eGFR or death. The primary outcome was the combined event. Renal survival referred to free of the combined events. The secondary outcomes were a 50% reduction of eGFR, ESRD, and the rate of renal function decline. Uncontrolled supportive care (USC) includes the management of hypertension, proteinuria, hyperlipidemia, nephrotic edema, and hypercoagulability.

### Statistical analysis

Categorical variables were expressed as frequencies plus percentages, and were compared by χ2 test or Fisher exact test. Continuous variables were expressed as medians and inter-quartile ranges, and compared by student-t test and Mann-Whitney *U* Test. The cumulative probability of renal survival was estimated by the Kaplan-Meier method, and compared with the Log-Rank test. Univariate and multivariate Cox regression models were used to evaluate the efficacy of different treatments and which variables affect renal survival. There were three adjusted models in multivariate Cox regression analyses. Variables using an enter method to entered into the multivariate regression models. Model one was adjusted for age, sex, MAP, eGFR, and proteinuria. Model two was adjusted for the variables in model one plus histological data. Model three was adjusted for the variables in model two plus RASB. *p* values were 2-tailed, and *p*-value < 0.05 was considered significant. All statistical analyses were performed using SPSS 26.0 (IBM).

## Results

### Patient characteristics

From July 2008 to December 2019, a total of 2724 biopsy-proven IgAN patients were initially screened. Eventually, 296 patients were included, and 44 patients received USC therapy, 164 patients received CS + CTX therapy, and 88 patients received CS + MMF therapy (Figure 1).

The median of age was 36.0 years. The median proteinuria value was 2068.5 mg/24 h. The median of serum creatinine was 163.5 μmol/L. The median of eGFR was 40.0 ml/min per 1.73 m^2^. Compared to the USC group, more patients with S1 and C1-2 in CS + CTX group and CS + MMF group. Compared to CS + CTX group, there were more patients with S1 in the CS + MMF group. Diastolic BP in the USC group were higher than other groups. MAP in the CS + MMF group was higher and C3 in the CS + CTX group was higher than the USC group. Other parameters at baseline were not significantly different among the three groups (Table 1).

**TABLE 1:** Baseline and follow-up characteristics of the study subjects.

Characteristic	All Patients (*n* = 296)	USC (*n* = 44)	CS + CTX (*n* = 164)	CS + MMF (*n* = 88)
Baseline (at Renal Biopsy)				
Age, years	36.0 (29.0, 48.8)	31.5 (26.0, 47.8)	38.5 (29.0, 48.0)	36.5 (28.0, 50.8)
Male, n (%)	194 (65.5)	32 (72.7)	104 (63.4)	58 (65.9)
Body weight, kg	68.8 (60.0, 78.0)	66.5 (59.3, 76.0)	68.3 (59.3, 77.8)	70.0 (60.0, 80.0)
Body mass index, kg/m^2^	24.0 (21.5, 27.3)	23.3 (22.0, 26.6)	24.1 (21.5, 27.6)	24.3 (21.3, 27.6)
Diabetes, n (%)	20 (6.8)	3 (6.8)	13 (7.9)	4 (4.5)
Systolic BP, mmHg	140.0 (127.3, 154.0)	140.0 (130.0, 159.0)	140.0 (124.3, 154.0)	138.0 (128.0, 151.5)
Diastolic BP, mmHg	90.0 (80.0, 100.0)	95.0 (81.8, 104.3)^#^	89.0 (79.3, 100.0)	86.0 (78.5, 95.0)
MAP, mmHg	106.7 (96.8, 116.3)	110.7 (100.6, 119.1)	104.8 (95.1, 117.2)	104.5 (94.5, 112.8)*
Prior immunosuppressive therapy, n(%)	5 (1.7)	0 (0.0)	3 (1.8)	2 (2.3)
Gross hematuria, n (%)	28 (9.5)	4 (9.3)	14 (8.5)	10 (11.5)
Proteinuria, mg/24 h	2068.5 (1513.5, 3059.5)	2135.0 (1481.3, 3007.5)	2028.0 (1487.0, 2860.0)	2250.0 (1583.3, 3574.0)
Proteinuria				
1–3 g/24h, n (%)	220 (74.3)	33 (75.0)	126 (76.8)	61 (69.3)
≥3 g/24h, n (%)	76 (25.7)	11 (25.0)	38 (23.2)	27 (30.7)
Serum creatinine, μmol/L	163.5 (137.6, 221.8)	170.3 (138.3, 237.0)	172.0 (138.0, 222.8)	158.0 (137.0, 207.5)
eGFR, mL/min per 1.73m^2^	40.0 (28.8, 50.2)	40.5 (27.7, 53.0)	38.3 (28.7, 48.7)	43.8 (32.2, 51.7)
CKD stage				
Stage 3, n (%)	213 (72.0)	32 (72.7)	113 (68.9)	68 (77.3)
Stage 4, n (%)	83 (28.0)	12 (27.3)	51 (31.1)	20 (22.7)
Serum albumin, g/dL	36.8 (31.4, 41.3)	36.0 (28.5, 40.2)	36.6 (32.2, 41.1)	37.6 (31.1, 41.9)
Serum IgA, g/L	2.7 (2.1, 3.7)	2.8 (2.2, 3.4)	2.7 (2.0, 3.7)	2.6 (2.2, 3.7)
C3, g/L	1.0 (0.9, 1.1)	0.9 (0.8, 1.0)	1.0 (0.9, 1.1)*	1.0 (0.9, 1.2)
Histological lesion scoring, n (%)				
M1	167 (63.7)	14 (56.0)	99 (63.5)	54 (66.7)
E1	79 (30.2)	6 (24.0)	49 (31.4)	24 (29.6)
S1	177 (67.6)	11 (44.0)^#^	102 (65.4)^#^	64 (79.0)^#^
T1-2	198 (75.6)	15 (60.0)	120 (76.9)	63 (77.8)
C1-2	124 (47.3)	6 (24.0)^#^	76 (48.7)	42 (51.9)
Treatment				
RASB, n (%)	203 (68.6)	24 (54.5)	116 (70.7)	63 (71.6)
Follow-up parameters and outcomes				
Follow-up, months	39.3 (23.8, 62.9)	30.5 (17.1, 50.2)	43.8 (28.2, 71.5)*	36.7 (21.6, 53.7)
Combined event, n (%)	125 (42.2)	29 (65.9)^#^	62 (37.8)	34 (38.6)
ESRD, n (%)	114 (38.5)	27 (61.4)^#^	59 (36.0)	28 (31.8)
≥50% reduction in eGFR, n (%)	113 (38.2)	28 (63.6)^#^	57 (34.8)	28 (31.8)
≥30% reduction in eGFR, n (%)	133 (44.9)	30 (68.2)^#^	67 (40.9)	36 (40.9)
TA-P, mg/24h	1485.0 (956.4, 2300.0)	1573.3 (1044.5, 2238.5)	1457.6 (900.2, 2329.7)	1541.1 (998.5, 2569.3)
TA-MAP, mmHg	103.4 (95.6, 113.7)	110.0 (101.7, 120.5)^#^	103.1 (95.0, 115.5)	101.2 (93.4, 110.2)
Rate of renal function decline, ml/min per 1.73 m^2^ per year	−2.4 (-8.8, 1.9)	−5.8 (-21.2, -2.5)^#^	−1.1 (-7.5, 2.3)	−1.5 (-8.8, 2.1)

USC, uncontrolled supportive care; CS, corticosteroids; CTX, cyclophosphamide; MMF, mycophenolate mofetil; BP, blood pressure; MAP, mean arterial pressure; eGFR, estimated glomerular filtration rate; CKD, chronic kidney disease; M1, mesangial hypercellularity; E1, endocapillary hypercellularity; S1, segmental glomerulosclerosis; T1, 26–50% tubular atrophy/interstitial fibrosis; T2, >50% tubular atrophy/interstitial fibrosis; C1, crescents in less than one fourth of glomeruli; C2, crescents in over one fourth of glomeruli; RASB, renin-angiotensin-aldosterone system blockers; TA-P, the time-average proteinuria; TA-MAP, time-average mean arterial pressure; ESRD, end stage renal disease; Combined event, eGFR ≥50%, ESRD, or death.

# *p* < 0.05, when compared with the rest two groups.

* *p* < 0.05, CS + MMF, or CS + CTX, vs. USC.

### Clinical outcomes

The median of follow-up period was 39.3 months (Table 1). One hundred and twenty-five (42.2%) patients experienced the combined event. The follow-up period in CS + CTX group was significantly longer than USC group. The incidence of ESRD, ≥ 30% reduction in eGFR, ≥ 50% reduction in eGFR, combined event was significantly higher in USC group than in the CS + CTX (*p* = 0.003, 0.002, *p* = 0.001 and *p* = 0.001, respectively) and CS + MMF group (*p* = 0.001, 0.005, 0.001and *p* = 0.005, respectively). The rate of renal function decline was significantly slower in the CS + CTX group (*p* < 0.001) and CS + MMF group (*p* = 0.001) than in the USC group [-5.8 ml/min per 1.73 m^2^ per year, IQR (-21.2, -2.5)]. TA-MAP was significantly higher in USC group than in the CS + CTX (*p* = 0.002) group and CS + MMF group (*p* < 0.001). The results showed no significantly difference between the CS + CTX group and CS + MMF group on outcomes.

**FIGURE 2:**
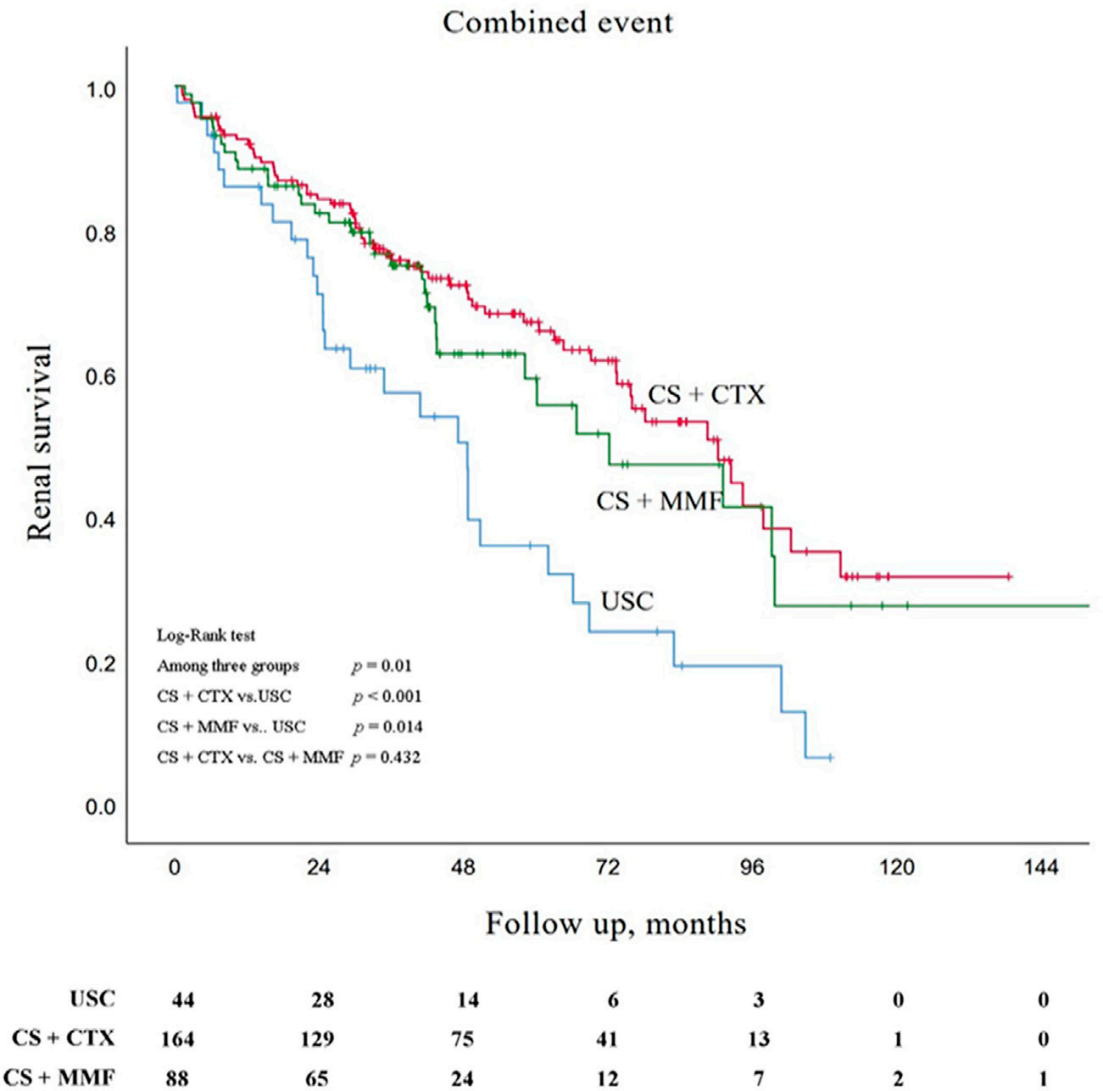
Kaplan-Meier survival curves for free of combined event in patients with uncontrolled supportive care (USC), corticosteroids (CS) combined with cyclophosphamide (CTX), and CS combined with mycophenolate mofetil (MMF).

**FIGURE 3:**
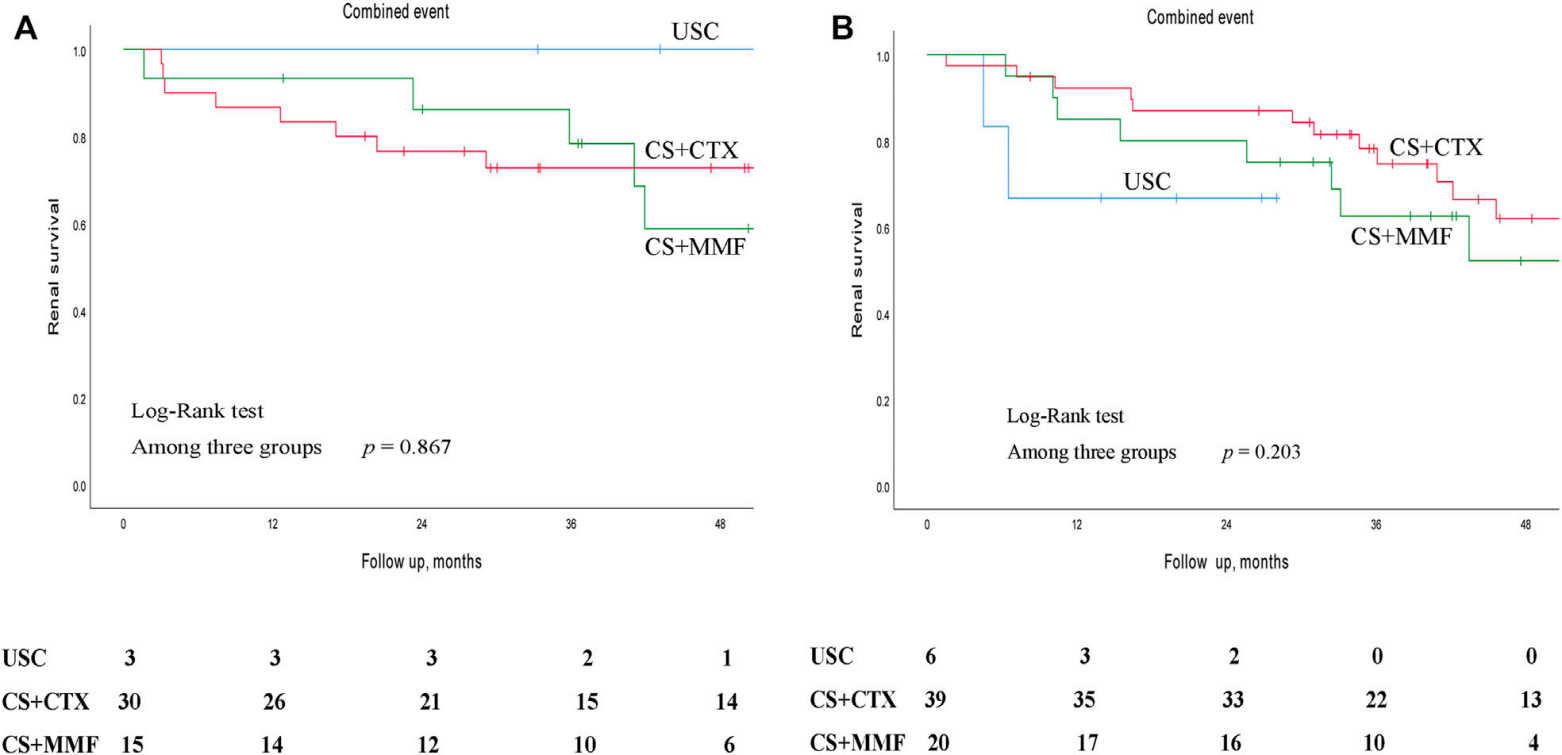
Kaplan-Meier survival curves for free of combined event of different subgroups in patients with uncontrolled supportive care (USC), corticosteroids (CS) combined with cyclophosphamide (CTX), and CS combined with mycophenolate mofetil (MMF). **(A)** In the subgroup of IgAN patients with E1 plus C1-C2 (regardless M, S, T). **(B)** In the subgroup of IgAN patients with S1 plus T1-T2 (with negative E and negative C).

Kaplan-Meier survival analysis showed that the estimated median time of renal survival in the USC, CS + CTX, and CS + MMF group were 48.6 months (95% CI 35.5–61.8 months), 90.3 months (95% CI 71.7–108.9 months), and 72.2 months (95% CI 37.0–107.3 months), respectively. The cumulative 5 - year renal survival rate of patients in the USC, CS + CTX, and CS + MMF group were 35.9, 67.1, and 59.2%, respectively. The cumulative 10 - year renal survival rate of patients in the USC, CS + CTX, and CS + MMF group were 6.4, 31.6, and 27.6%, respectively. Compared to the USC group, CS + CTX group and CS + MMF group had a significantly higher rate of renal survival (*p* < 0.001 and *p* = 0.014, respectively) (Figure 2). Even though the 5-year and 10-year renal survival rate in the CS + CTX group were both higher than the CS + MMF group, CS + CTX therapy has a trend of better renal survival, but the results showed that there is no significant difference (*p* = 0.432) (Figure 2). Further, survival analysis showed that the renal survival rate had no statistical differences among USC, CS + CTX, and CS + MMF groups for the subgroup of IgAN patients with E1 plus C1-2 (regardless M, S, T) and the subgroup of IgAN patients with S1 plus T1-2 (with negative E and negative C) (*p* = 0.867 and *p* = 0.203, respectively) (Figures 3A,B).

We used univariate and multivariate Cox regression analyses to ensure the independent significance of different treatments for the combined event (Table 2). Univariate Cox regression analysis showed that CS + CTX therapy (HR = 0.439, 95% CI 0.282 - 0.684, *p* < 0.001) and CS + MMF therapy (HR = 0.527, 95% CI 0.320 - 0.869, *p* = 0.012) both significantly reduced the incidence of combined event when compared with the USC. Further, we created three multivariate Cox regression models, shown in Table 2. The results showed that CS + CTX therapy (HR = 0.457, 95% CI 0.238 - 0.878, *p* = 0.019) significantly reduced the incidence of combined event after adjusted for age, sex, MAP, eGFR, proteinuria, M1, E1, S1, T1-2, C1-2 and RASB, but CS + MMF therapy (HR = 0.523, 95% CI 0.246 - 1.109, *p* = 0.091) did not reduce the incidence of combined event after adjusted for age, sex, MAP, eGFR, proteinuria, M1, E1, S1, T1-2, C1-2 and RASB. Similar results were also shown in the other two models.

**TABLE 2:** Predictors of combined events in IgA nephropathy patients with stage 3 or four CKD.

	USC	CS + CTX		CS + MMF	
		HR (95% CI)	*p* Value	HR (95% CI)	*p* Value
Univariate	Ref	0.439 (0.282, 0.684)	<0.001	0.527 (0.320, 0.869)	0.012
Multivariate model 1^a^	Ref	0.504 (0.320, 0.793)	0.003	0.672 (0.400, 1.127)	0.132
Multivariate model 2^b^	Ref	0.512 (0.268, 0.976)	0.042	0.638 (0.305, 1.332)	0.231
Multivariate model 3^c^	Ref	0.457 (0.238, 0.878)	0.019	0.523 (0.246, 1.109)	0.091

a Model 1 was adjusted for age, sex, MAP, proteinuria, and eGFR.

b Model 2 was adjusted for age, sex, MAP, proteinuria, eGFR, M1, E1, S1, T1-2, C1-2.

c Model 3 was adjusted for age, sex, MAP, proteinuria, eGFR, M1, E1, S1, T1-2, C1-2 and RASB.

### Adverse events

The incidence of pneumonia in the CS + MMF group was significantly higher than USC group (9.1 vs. 0.0%, *p* = 0.039), there was no statistical difference with the CS + CTX group (9.1 vs. 3.7%, *p* = 0.073). The incidence of death due to infection in CS + MMF group was significantly higher than CS + CTX group (5.7 vs. 1.2%, *p* = 0.040), and also higher than the USC group (5.7 vs. 0.0%), but this was not statistically significant (*p* = 0.107). For other adverse event, no statistic difference was observed among the three groups (Table 3).

**TABLE 3:** Adverse events in IgA nephropathy patients with moderate renal insufficiency.

	All Patients (*n* = 296)	USC (*n* = 44)	CS + CTX (*n* = 164)	CS + MMF (*n* = 88)	*p* Value
Pneumonia, n (%)	14 (4.7)	0 (0.0)	6 (3.7)	8 (9.1)*	0.043
Leukopenia, n (%)	89 (30.1)	10 (22.7)	51 (31.1)	28 (31.8)	0.512
Anemia, n (%)	56 (18.9)	8 (18.2)	30 (18.3)	18 (20.5)	0.908
Stomach discomfort, n (%)	16 (5.4)	1 (2.3)	6 (3.7)	9 (10.2)	0.054
Hepatic injury, n (%)	51 (17.2)	4 (9.1)	35 (21.3)	12 (13.6)	0.091
Diabetes mellitus, n (%)	15 (5.1)	3 (6.8)	9 (5.5)	3 (3.4)	0.656
Low back pain, n (%)	9 (3.0)	0 (0.0)	4 (2.4)	5 (5.7)	0.160
Tumor, n (%)	3 (1.0)	0 (0.0)	2 (1.2)	1 (1.1)	0.766
Death due to pneumonia, n (%)	7 (2.4)	0 (0.0)	2 (1.2)	5 (5.7)^#^	0.045
Total death, n (%)	10 (3.4)	1 (2.3)	3 (1.8)	6 (6.8)	0.102

CS, corticosteroids; CTX, cyclophosphamide; MMF, mycophenolate mofetil; UCS, uncontrolled supportive care.

# , *p* < 0.05, CS + MMF, vs. CS + CTX.

* , *p* < 0.05, CS + MMF, vs. USC.

Ten patients (3.4%) died due to any cause (Table 3), but there was no statistical difference among the three groups (Table 3). Seven patients (2.4%) death due to severe pneumonia, five of eight (75%) patients in the CS + MMF group and two of six (33.3%) patients in the CS + CTX group with severe pneumonia died from severe pneumonia, respectively. One patient in the USC group died due to traffic accident. One patient in the CS + CTX group and one patient in CS + MMF group died due to cardiovascular disease.

## Discussion

As our best knowledge, our present study is the first study compared the efficacy and safety of low-dose CS combined with CTX or MMF therapy for IgAN patients with stage 3 or 4 CKD and proteinuria ≥1.0 g/24 h. Our findings supported that compared with the USC group, the CS + CTX therapy and the CS + MMF therapy significantly reduced the risk of the combined event, ESRD, a ≥50% reduction in eGFR and slowed down the rate of renal function decline in IgAN patients with stage 3 or 4 CKD and proteinuria ≥1.0 g/24 h, but no significant difference between the CS + CTX group and the CS + MMF group was found. However, the incidence of pneumonia and death due to infection in the CS + MMF group was higher than the other two groups. Thus, present study showed that CS + CTX therapy maybe better option for IgAN patients with stage 3 or 4 CKD.

The CS + CTX therapy and the CS + MMF therapy were both effective for IgAN patients with stage 3 or 4 CKD and severe proteinuria. Since there are no established treatment guidelines for IgAN patients with renal insufficiency, therapeutic regimens are mainly determined by doctor’s experience. The clinicians tend to give IgAN patients with renal insufficiency steroid-based immunosuppressive therapy based on the following practical significances. First, IgAN often occurs in young adults (Lai et al., 2016), and nearly 20% of IgAN patients in Chinese were accompanied with renal insufficiency at biopsy. These patients often progressed to ESRD within 7–10 years if they only received supportive care therapy (Lv et al., 2008). Our previous study showed that more than 60% of IgAN patients with stage 3 or 4 CKD and severe proteinuria at biopsy progressed to ESRD after 3 years follow-up period if they received USC therapy. A study in Italy demonstrated that IgAN was the first cause that led to dialysis in people less than 40 years old (Pozzi et al., 2016). Second, although the pathogenesis of IgAN is incompletely understood, it is well recognized as an autoimmune mediated glomerulonephritis characterized by the deposition of IgA-dominant or co-dominant immune complex in the glomeruli (Roberts, 2014). These immune complexes are nephritogenic and directly contribute to glomerular inflammation (Rodrigues et al., 2017). The immunologic mechanisms in the development and progression of IgAN make immunosuppressive therapy a reasonable option (Roberts, 2014). Steroids worked primarily through anti-inflammatory effects by inhibiting the genes expression of inflammatory cytokines (Lai et al., 2016). Immunosuppressants treat and block the formation of immune complexes and inflammatory reactions in renal tissue through inhibiting the proliferation of B lymphocytes and further reducing antibody synthesis (Allison and Eugui, 2000). Third, immunosuppressants were commonly used in IgAN patients with renal insufficiency in clinical practice. The VALIGA study which contained 1147 IgAN patients showed that immunosuppressants were more frequently used in IgAN patients with eGFR less than 30 ml/min per 1.73 m^2^ than in those with eGFR more than 30 ml/min per 1.73 m^2^ (66 vs 44%, *p* = 0.004) (Coppo et al., 2014). Therefore, steroid-based immunosuppressive therapy was widely subscribed to IgAN patients with renal insufficiency. The dose of immunosuppressants should be reduced in elderly patients or patients with renal insufficiency to reduce the incidence of adverse events. Elderly patients often associated with impaired organ function, affecting the metabolism and excretion of immunosuppressants, need reduced the dose of immunosuppressants (Kant et al., 2022). Also, the dose of cyclophosphamide should be reduced (by ≥ 30%) in patients with eGFR <30 ml/min per 1.73 m^2^ (Kidney Disease: Improving Global Outcomes Glomerular Diseases Work, 2021). Our present results showed that the CS + CTX therapy and the CS + MMF therapy both significantly reduced the risk of the combined event and protected the renal function for IgAN patients with stage 3 or 4 CKD, which was consistent with previous studies which contained IgAN patients with renal insufficiency (Roccatello et al., 2000; Ballardie and Roberts, 2002; Chen et al., 2002; Tang et al., 2010; Ma et al., 2020; Zhao et al., 2021; Beck et al., 2022).

More importantly, this study showed that the CS + CTX therapy seems more effective than the CS + MMF therapy. There are a variety of steroid-based immunosuppressive therapies. For IgAN patients with renal insufficiency, the CS + CTX therapy or the CS + MMF therapy were the most common treatment therapies in our hospital. Results showed that the CS + CTX therapy and the CS + MMF therapy both effectively improved renal prognosis. However, whether the CS + CTX therapy is more effective or the CS + MMF therapy is still unclear in this context. Our results showed there is no significant difference between the CS + CTX group and the CS + MMF group on primary and secondary outcomes. The Kaplan-Meier survival analysis showed that the renal survival rate in the CS + CTX group was higher than in the CS + MMF group despite no statistical difference identified. Meanwhile, multivariate Cox regression showed that CS + CTX therapy independently reduced the incidence of the combined event after adjusted for age, sex, MAP, eGFR, proteinuria, M1, E1, S1, T1-2, C1-2, and RASB, but the CS + MMF therapy did not, which means the CS + CTX therapy not the CS + MMF therapy was an independent protective factor of the combined event. Taken together, it may be indicated that the CS + CTX therapy was more effective than the CS + MMF therapy.

Safety evaluations in present study indicated that the CS + CTX therapy was more safety than the CS + MMF therapy. Our study showed the incidence of severe pneumonia was 9.1% in the CS + MMF group. Inconsistently, the incidence of severe pneumonia was 2.2% in the CS monotherapy group of the TESTING trial (Lv et al., 2017), 3.7% in the immunosuppression group of the STOP-IgAN trial (Rauen et al., 2015), and 3.4% in MMF plus prednisone group of Hou et al.’ study (Hou et al., 2017). There are three potential reasons for this inconsistency. Firstly, the patients in RCT were almost IgAN patients with normal renal function, but in our present study were IgAN patients with renal insufficiency. Secondly, MMF is mainly metabolized into mycophenolic acid phenyl glucuronide (MPAG), eliminated primarily through renal excretion (Johnson et al., 1998). With a decline in renal function, urea and other uremic compounds accumulate, which would compete for albumin binding sites, leading to the reduction in renal clearance of MPAG (Tornatore et al., 2015). Thus, MMF accumulates in IgAN patients with renal insufficiency (referring to stage 3 or 4 CKD). Thirdly, monitoring the appearance of adverse events during the follow-up period was rigorous in RCT, patients can be taken good care and doctors can give patients with infection prompt treatment, so most patients with infection would not progress to severe pneumonia. But in our cohort, most patients only come to the hospital when they are already had severe pneumonia, treatment was belated. However, the incidence of pneumonia was 3.7% in the CS + CTX group, which was consistent with 3.7% of the STOP-IgAN trial. The incidence of pneumonia and death due to pneumonia was more in the CS + MMF group, and the incidence of other adverse events was similar between the CS + CTX group and the CS + MMF group. Overall, CS + CTX therapy was safer than CS + MMF therapy.

Our study has several limitations. First, the single-center retrospective nature of our study may imply a lower level of evidence and inevitable selection bias, therefore, the results on efficacy and safety between these three groups should be considered as not definitive. However, the relatively low rate of loss to follow-up in our cohort increased the reliability of our results. Second, due to the related adverse events of RASB for IgAN patients with renal insufficiency, only 68.6% of the included patients used RASB as one of the supportive care therapies, but there is no significant difference among the three groups. Third, all IgAN patients included in our present study were Chinese, so our findings cannot easily translate to non-Asian IgAN patients.

## Conclusion

Compared to the use of CS + CTX and CS + MMF in IgAN patients with stage 3 or 4 CKD and severe proteinuria could significantly increase the risk of renal survival and attenuate the rate of renal function decline. The renal survival in CS + CTX group seems better than that in CS + MMF group. The incidence of pneumonia and death due to infection in CS + MMF group was higher than the other groups. Multicenter prospective randomized controlled trials with larger sample size are needed to verify our findings.

## Data Availability

The raw data supporting the conclusion of this article will be made available by the authors, without undue reservation.
